# A prospective compound screening contest identified broader inhibitors for Sirtuin 1

**DOI:** 10.1038/s41598-019-55069-y

**Published:** 2019-12-20

**Authors:** Shuntaro Chiba, Masahito Ohue, Anastasiia Gryniukova, Petro Borysko, Sergey Zozulya, Nobuaki Yasuo, Ryunosuke Yoshino, Kazuyoshi Ikeda, Woong-Hee Shin, Daisuke Kihara, Mitsuo Iwadate, Hideaki Umeyama, Takaaki Ichikawa, Reiji Teramoto, Kun-Yi Hsin, Vipul Gupta, Hiroaki Kitano, Mika Sakamoto, Akiko Higuchi, Nobuaki Miura, Kei Yura, Masahiro Mochizuki, Chandrasekaran Ramakrishnan, A. Mary Thangakani, D. Velmurugan, M. Michael Gromiha, Itsuo Nakane, Nanako Uchida, Hayase Hakariya, Modong Tan, Hironori K. Nakamura, Shogo D. Suzuki, Tomoki Ito, Masahiro Kawatani, Kentaroh Kudoh, Sakurako Takashina, Kazuki Z. Yamamoto, Yoshitaka Moriwaki, Keita Oda, Daisuke Kobayashi, Tatsuya Okuno, Shintaro Minami, George Chikenji, Philip Prathipati, Chioko Nagao, Attayeb Mohsen, Mari Ito, Kenji Mizuguchi, Teruki Honma, Takashi Ishida, Takatsugu Hirokawa, Yutaka Akiyama, Masakazu Sekijima

**Affiliations:** 1 Education Academy of Computational Life Sciences (ACLS), Tokyo Institute of Technology, 4259 Nagatsutacho, Midori-ku, Yokohama, 226-8501 Japan; 2 Department of Computer Science, School of Computing, Tokyo Institute of Technology, 2-12-1 Ookayama, Meguro-ku, Tokyo, 152-8550 Japan; 3 Advanced Computational Drug Discovery Unit, Tokyo Institute of Technology, J3-23-4259 Nagatsutacho, Midori-ku, Yokohama, 226-8501 Japan; 4 RIKEN Medical Sciences Innovation Hub Program, 1-7-22, Suehiro-cho, Tsurumi-ku, Yokohama, 230-0045 Japan; 5 Bienta/Enamine Ltd., 78 Chervonotkatska Street 78, Kyiv, 02094 Ukraine; 6 Faculty of Pharmacy, Keio University, 1-5-30 Shibakoen, Minato-ku, Tokyo, 105-8512 Japan; 7 Research Fellow of the Japan Society for the Promotion of Science DC1, Tokyo, Japan; 8 Department of Biological Science, Purdue University, West Lafayette, Indiana, 47907 USA; 9 Department of Computer Science, Purdue University, Indiana, 47907 USA; 10 Department of Biological Sciences, Chuo University, 1-13-27 Kasuga, Bunkyo-ku, Tokyo, 112-8551 Japan; 11 Discovery technology research department, Research division, Chugai Pharmaceutical Co.,Ltd., 200, Kajiwara, Kamakura, Kanagawa 247-8530 Japan; 12 Okinawa Institute of Science and Technology Graduate University, 1919-1 Tancha, Onna-son, Kunigami, Okinawa, 904-0495 Japan; 13 The Systems Biology Research Institute, Falcon Building 5F, 5-6-9 Shirokanedai, Minato-ku, Tokyo, 108-0071 Japan; 14 Center for Integrative Medical Sciences, RIKEN, 1-7-22 Suehiro-cho, Tsurumi-ku, Yokohama City, Kanagawa 230-0045 Japan; 15 Graduate School of Humanities and Sciences, Ochanomizu University, 2-1-1 Otsuka, Bunkyo-ku, Tokyo, 112-8610 Japan; 16 Department of Computational Biology and Medical Sciences, Graduate School of Frontier Sciences, The University of Tokyo, 7-3-1 Hongo, Bunkyo-ku, Tokyo, 113-8654 Japan; 17 Center for Simulation Science and Informational Biology, Ochanomizu University, 2-1-1 Otsuka, Bunkyo-ku, Tokyo, 112-8610 Japan; 18 School of Advanced Science and Engineering, Waseda University, 3-4-1 Okubo, Shinjuku-ku, Tokyo, 169-8555 Japan; 19 IMSBIO Co., Ltd., Level 6 OWL TOWER, 4-21-1 Higashi-Ikebukuro, Toshima-ku, Tokyo, 170-0013 Japan; 20 Department of Biotechnology, Bhupat Jyoti Mehta School of Biosciences, Indian Institute of Technology Madras, Chennai, 600036 Tamilnadu India; 21 CAS in Crystallography and Biophysics and Bioinformatics Facility, University of Madras, Chennai, 600025 Tamilnadu India; 22 Okazaki City Hall, 2-9 Juo-cho Okazaki, Aichi, 444-8601 Japan; 23 IQVIA Services Japan K.K., 4-10-18 Takanawa Minato-ku, Tokyo, 108-0074 Japan; 24 Institute for Chemical Research, Kyoto University, Uji, Kyoto, 611-0011 Japan; 25 Department of Chemistry & Biotechnology, The University of Tokyo, 7-3-1 Hongo, Bunkyo-ku, Tokyo, 113-8654 Japan; 26 Biomodeling Research Co., Ltd., 1-704-2 Uedanishi, Tenpaku-ku, Nagoya, 468-0058 Japan; 27 Faculty of Medicine, Akita University, 1-1-1 Hondo, Akita, 010-8543 Japan; 28 Isotope Science Center, The University of Tokyo, 2-11- 16, Yayoi, Bunkyo-ku, Tokyo, 113-0032 Japan; 29 Department of Biotechnology, The University of Tokyo, 1-1-1 Yayoi, Bunkyo-ku, Tokyo, 113-8657 Japan; 30 Google Japan Inc., 6-10-1 Roppongi, Minato-ku, Tokyo, 106-6126 Japan; 31 Department of Computational Science and Engineering, Nagoya University, Furocho, Chikusa-ku, Nagoya, 464-8603 Japan; 32 Tosei General Hospital, 160 Nishioiwake-cho, Seto, Aichi 489-8642 Japan; 33 Department of Complex Systems Science, Graduate School of Information Science, Nagoya University, Furocho, Chikusa, Nagoya, 464-8601 Japan; 34 National Institutes for Biomedical Innovation, Health and Nutrition, Osaka, 567-0085 Japan; 35 RIKEN Center for Biosystems Dynamic Research, 1-7-22 Suehiro-cho, Tsurumi-ku, Yokohama, Kanagawa 230-0045 Japan; 36 Molecular Profiling Research Center for Drug Discovery, National Institute of Advanced Industrial Science and Technology, 2-4-7 Aomi, Koto-ku, Tokyo, 135-0064 Japan; 37 Division of Biomedical Science, Faculty of Medicine, University of Tsukuba, 1-1-1 Tennodai, Tsukuba-shi, Ibaraki, 305-8575 Japan; 38 Initiative for Parallel Bioinformatics, Level 14 Hibiya Central Building, 1-2-9 Nishi-Shimbashi Minato-Ku, Tokyo, 105-0003 Japan; 39 Training Program of Leaders for Integrated Medical System (LIMS), Kyoto University, Sakyo-ku, Kyoto, 606-8501 Japan; 40 Present Address: Otemachi Bldg. 3F, 1-6-1, Preferred Networks, Otemachi, Chiyoda-ku, Tokyo, 100-0004 Japan

**Keywords:** Virtual drug screening, Information technology

## Abstract

Potential inhibitors of a target biomolecule, NAD-dependent deacetylase Sirtuin 1, were identified by a contest-based approach, in which participants were asked to propose a prioritized list of 400 compounds from a designated compound library containing 2.5 million compounds using *in silico* methods and scoring. Our aim was to identify target enzyme inhibitors and to benchmark computer-aided drug discovery methods under the same experimental conditions. Collecting compound lists derived from various methods is advantageous for aggregating compounds with structurally diversified properties compared with the use of a single method. The inhibitory action on Sirtuin 1 of approximately half of the proposed compounds was experimentally accessed. Ultimately, seven structurally diverse compounds were identified.

## Introduction

In the early stages of the drug discovery process, active compounds, inhibitors and/or activators, for a target biomolecule are sought. The high-throughput screening (HTS) campaign can be experimentally used to identify as many active compounds as possible from a compound library. However, the odds of finding active compounds using HTS is low. A previous study concluded that the success rate ranges from 0.05% to 0.14% depending on the class of target biomolecule (kinase, GPCR, protein-protein interaction, etc.)^1^. To improve odds, computer-aided drug discovery or virtual screening (VS) methods have been employed^2,3^.

VS methods utilize structural information of a target biomolecule and/or experimentally known actives/inactives. The former is known as structure-based VS (SBVS), and the latter, as ligand-based VS (LBVS). The representative of SBVS is docking study, which docks test compounds to a modeled target biomolecule. There are many parameters that are used in docking studies to determine the ranking of test compounds, e.g., how the target biomolecule is modeled, whether the target biomolecules are treated as a rigid body or a flexible body, how the compounds of interest are oriented in relation to the target molecule, and how the score of the placed compound is defined^4,5^. LBVS is based on a hypothesis that test compounds with similar properties of known actives would have similar activities. To rank the test compounds, a simple comparison of the similarity of the chemical structure or structure-activity relationship (SAR) using actives and inactives is performed^6^. Combining LBVS and SBVS is also common^2^.

Performance comparisons of various VS methods have been addressed using known active/inactive compounds; in other words, benchmarks have been conducted retrospectively^7^. Such benchmark results have led to obscure outcomes when VS methods are used in a prospective way. In addition, the performance of VS methods differs under various conditions, and no set standard exists^8^. Hence, currently we are not supplied with enough knowledge for choosing a suitable method for a designated compound library and target biomolecule before experimental validation.

Utilizing various VS methods for a certain problem is an alternative approach for relying on a single VS approach. For the various methods, we conducted two compound screening contests, in which participants were asked to propose potential active compounds of a target biomolecule from commercially available 2.2- and 2.4-million compound lists. The inhibitory functions of the proposed compounds were experimentally assessed. In the first and second contests held in 2014^9^ and 2015^10^, respectively, we identified several hit compounds with inhibitory activity of the target molecule, *i.e*., the tyrosine-protein kinase Yes. Participants employed various methods. In the second contest, we found that iterating the contest with the same target would give improved hit rates and enable identification of the statistically warranted method.

To verify robustness of the concept of the contest-based approach, we conducted another contest in 2016 employing a new target biomolecule, nicotinamide adenine dinucleotide (NAD)-dependent deacetylase Sirtuin 1, hereafter referred to as Sirtuin 1. Sirtuin 1 is a member of the sirtuin family (Sirtuin 1 to Sirtuin 7). The structure of Sirtuin 1 changes from an open state to a closed state upon the addition of cofactor (NAD^+^) and a substrate, and both open and closed structures have been reported (PDBID: 4IG9, 4KXQ)^11^. Other relevant structural information is also available (see Table 1). Information of actives and inactives has been deposited in open databases, such as BindingDB^12,13^ and ChEMBL^14^. Histone deacetylase (HDAC) inhibitors and decoys were previously compiled in the MUBD-HDACs database, in which information of Sirtuin 1 is included^15^. Hence, SBVS and LBVS can be employed for the target.

**Table 1:** Summary of the methods used by participating groups.

Group	Modeling of Sirtuin 1 structure		Ligand preparation	Processing method of compound library		
	3D structure modeling/prediction methods/tools	PDB ID used		Filter class	Actives	Decoys
1	—	4KXQ^11^	*OMEGA*^61^	LB→SB	Cambinol, HR73, salermide, sirtinol, suramin, and tenovin	
2	HM (*FAMS*)^21^	4BN5^20^	*Open babel*^62^	LB→SB	Cocrystalized ligands in PDB and ChEMBL (IC_50_ < 1 μM)	—
3	—	—	*PaDEL-Descriptor*^23^	LB	MUBD-HDACs^63^	
4	—	4KXQ	*CORINA*^64^	Hybrid (LB&SB)	PubChem (CID703333, CID71459392)	—
5	—	4ZZJ^65^	*Open babel*. *Dock*^27^	Hybrid (LB&SB)	Known Sirtuin inhibitors	—
6	—	—	*RDKit*^66^	LB	ChEMBL (CHEMBL4506, CHEMBL4462, CHEMBL4461), PubChem (AID 652115), BindingDB (Target = NAD-Dependent Deacetylase Sirtuin 1)	
7		4IG9,^11^ 4IF6, 4ZZI,^65^ 4I5I,^33^ 4ZZJ	*Ligprep*^67^	Hybrid (LB&SB)	Cocrystalized ligands	
8	MD (*myPresto*, *cosgene*^37^)	4ZZI	*myPresto (AM1, AM1BCC)*	Hybrid (LB → SB)	Selisistat (EX-527), Compound 28^35^	—
9	HM and minimization (*SWISS model*, *Foldit*)	4ZZI	*Open babel, Discovery Studio visualizer*	SB		
10	HM (*Prime*)^41,42^	4ZZI (selected from 4I5I, 4IF6, 4IG9, 4KXQ, 4ZZI, 4ZZJ, 5BTR^68^)	*LigPrep*	Hybrid (LB&SB)	ChEMBL (IC_50_ < 20 μM)	ChEMBL (IC_50 _> 100 μM)
11	*myPresto* (*tplgeneX*)^37,38^	4I5I	*myPresto* (*create3D*)	SB	EX-527 analogue^33^	DUD-E^8^
12	—	—	*RDKit*	LB	BindingDB	ZINC^47^
13	MD	4I5I	*OMEGA*	Hybrid (LB, SB&visual inspection)	Sun *et al*.^49^	—
14	—	4ZZI	*LigandBOX*^52^	Hybrid (LB & SB)	8 compounds including Splitomicin, Cambinol, Salerminde	—
15	—	4I5I	*OMEGA*	Hybrid (LB&SB)	Cocrystalized ligands in PDB (4I5I, 4ZZI, 4IF6)	—
16		4ZZI		Hybrid (LB → SB)		

Software names are given in italic.

PDB = Protein Data Bank; LB = ligand-based; SB = structure-based; HM = homology modeling; MD = molecular dynamics simulation.

The third compound screening contest was organized by the Initiative for Parallel Bioinformatics (IPAB). The submission period of compound proposals started in January 20, 2016, and ended in May 20, 2016. Sixteen groups participated in the contest. The participants were asked to propose a prioritized set of 400 compounds. We selected approximately 200 compounds from each group and, in total, 3,192 unique compounds were assayed. Seven potent compounds with half-maximal inhibitory concentrations (IC_50_) less than 20 μM were identified. The benefits of collecting proposed compounds via various methods are discussed in the following.

## Methods

### Preparation of the compound library

To create a compound library for the contest, we obtained a compound library from Enamine Ltd., which listed 2,459,912 available compounds in their inventory. We searched this inventory for compounds that were reported to interact or not interact with Sirtuin 1 to 7 in BindingDB^12,13^ and ChEMBL version 20^16^. We found 1,443 unique compounds, among which 44 compounds in the compound library were eliminated. Finally, the compound library used in the contest contained 2,459,868 compounds, which was distributed to the participants of the contest.

### Methods used by the participants

Sixteen groups participated in the contest, proposing various methods as shown in Table 1. Proposed compounds in canonical SMILES format with prioritized ranking are given in the supporting materials. Here, we briefly describe each method.

#### Group 1 (G1)

Compounds in the library were first filtered by two-dimensional (2D) similarity with six known drugs for the target using SIMCOMP^17^. The top 20% of similar compounds were selected and used for structure-based virtual screening. The human Sirtuin 1 crystal structure (PDBID: 4KXQ) was used as a receptor. From the prescreened compound library, PL-PatchSurfer2^18^ was used to select the top 2,000 molecules. These molecule were docked to Sirtuin 1 by AutoDock Vina^19^. The compounds were ranked by a sum of Z-scores from each program.

#### Group 2 (G2)

Compounds in the library were filtered by the Tanimoto coefficient of 0.8 for the active chemicals detected in ChEMBL in relation to the target protein (query: SIR1_HUMAN). The protein structure (PDBID: 4BN5^20^), containing cocrystalized coenzyme carba-nicotinamide-adenine-denucleotide (CNA) and ligand N-[2-[3-(piperazin-1-ylmethyl)imidazo[2,1-B][1,3]thiazol-6-yl]phenyl]quinoxaline-2-carboxamide (SR7), was used to model a template protein with the FAMS program^21^. An *in silico* screening approach, ChooseLD^22^, was applied to the model. Both the original version and the hydrophobic interaction, including a version of ChooseLD, were used in the presence or absence of Coenzyme CNA. Then, four kinds of ChooseLD patterns were determined, among which highly ranked compounds were equally selected.

#### Group 3 (G3)

An SAR model was trained by utilizing Sirtuin 1 and HDAC inhibitors and decoys from the MUBD-HDACs database.^15^ In detail, 770 1D and 2D descriptors and 881 PubChem fingerprints through the PaDEL descriptor^23^ for compounds were generated. The extremely random tree algorithm^24^ was employed for learning SAR models. Next, a trained model was applied to the compound library to predict the compound activity. Finally, the top-ranked 400 compounds were proposed for further validation based on the predicted score.

#### Group 4 (G4)

A series of rational methods was applied to screen the compound library against the target protein. The methods included SAR analysis, docking simulation, database mining, substructure searching and empirical inspection. For structure-based studies, a post refined protein model originally retrieved from PDB (PDBID: 4KXQ) was applied as a target structure. Multiple docking tools were used in the screening. Machine learning methods^25,26^ were then applied to assess the binding potentials and to identify the most predictive binding mode that originated from those docking tools.

#### Group 5 (G5)

In total, 110,000 compounds were chosen from the compound library based on their structural similarity to known Sirtuin 1 inhibitors and Lipinski’s rule of five. To further screen the compounds, the complex structures of Sirtuin 1 (PDBID: 4ZZJ) with the selected compounds were modeled, and the grid scores were obtained by docking calculations with Dock6 program code^27^. A new measure named the Ligand Triangle (LT) score was also introduced, and the candidate compounds were ranked according to the square sum of the two scores.

#### Group 6 (G6)

Assay data of not only Sirtuin 1 but also its paralogous enzymes were utilized to train a regression model using a transfer learning method. Here, activity values of the training data were standardized to inhibition rates at 20 μM using Hill’s equation^28^ under the assumption that the Hill coefficient is 1. Compounds with similar physicochemical properties to those of known inhibitors were extracted from the library using a modified method of QED^29,30^. Inhibition rates of these compounds were predicted with the trained model^24^. Then, compounds that were predicted to possess the highest inhibition rates were proposed.

#### Group 7 (G7)

Structure- and pharmacophore-based approaches were used to identify Sirtuin 1 inhibitors. In the structure-based method, open (PDBID: 4IG9), closed (PDBID: 4IF6), and liganded (PDBID: 4ZZI, 4I5I, 4ZZJ) conformations were used, and 500 compounds were shortlisted from the compound library. Clustering yielded 300 compounds with chemical diversity. In the pharmacophore-based approach,^31,32^ the cocrystallized structures of Sirtuin 1 with an inhibitor and a substrate were used to derive a pharmacophore with the features and the geometry suitable to bind the active site of Sirtuin 1. Hit-libraries that resulted from structure-based screenings were screened again using the pharmacophore to obtain 100 compounds with the best fitness. In total, 400 compounds were submitted.

#### Group 8 (G8)

Among the compound library, compounds that possess an amide or thioamide group bound to a ring, lactams and thiolactams were first extracted, considering previous SAR studies^33–35^. Then, nondrug-like compounds and smaller or larger compounds were excluded. A molecular dynamics (MD) simulation of the structure of Sirtuin 1 (PDB ID: 4ZZI) was performed, and multiple coordinates were obtained from the MD trajectory. myPresto^36–38^ and a cloud computing environment was utilized for the docking simulation. The results were scored by the multiple target screening (MTS) method.^36^ Finally, inappropriate structures were eliminated by visual inspection.

#### Group 9 (G9)

An initial 3D structure of Sirtuin 1 was prepared by the SWISS model using the structure of Sirtuin 1 (PDBID: 4ZZI) as a modeling template^39^. To clean up structural error in the prepared model, sidechain structures were optimized by Foldit standalone version^40^. To reduce calculation costs, the compounds in the library were filtered by human inspection considering the Sirtuin 1 binding site condition. The filtered compounds were evaluated by docking simulation (AutoDock Vina^19^ with PyRx 0.8.). Finally, possibly favorable compounds were selected by visual inspection and scored by AutoDock Vina.

#### Group 10 (G10)

Homology modeling by Prime^41,42^ was used to obtain the target protein structure. Seven homology models were evaluated by decoy docking of 122 known active compounds and 200 inactive compounds obtained from the ChEMBL database. Compounds in the library with a molecular weight less than 500 were screened by Glide SP mode docking^43,44^. Finally, the top 50,000 compounds were reranked by the SIEVE-Score,^45^ and the similarity of the interaction energy between a compound and each amino acid residue compared with the known active and decoy compounds was evaluated.

#### Group 11 (G11)

A Sirtuin 1 structure (PDBID: 4I5I) was chosen to carry out the docking simulation. 4I5I contains a ligand that inhibits Sirtuin 1 activity (EX-527).^33^ Therefore, the place where the ligand was bound attracted our attention, and our group attempted to find molecules that could bind at that location. Ligand efficiencies of the compounds in the library were calculated using the myPresto system^37,38^. Then, after eliminating optical isomers, the top 400 compounds that had the highest ligand efficiency were chosen as molecules with inhibitory activity.

#### Group 12 (G12)

RankSVM,^46^ a machine learning method for ranking prediction, was used to construct the prediction model. Training data consisted of two parts. One part comprised samples of compounds with an IC_50_ report of Sirtuin 1, 2 and 3 in BindingDB. The other part comprised random samples from ZINC^47^ as pseudo inactive compound data. ECFP4^48^ fingerprint was used as a feature vector. The cost parameter of RankSVM was chosen from 2^−17,^2^−15^, …, 2^15^ with 5-fold cross validation. The evaluation was based on the top-*k* version of normalized discounted cumulative gain (NDCG) with a *k* of 100.

#### Group 13 (G13)

First, LBVS was performed for the compound library using known actives described by Sun *et al*.^49^ Then, the output compounds from LBVS were applied to ensemble-docking using Sirtuin 1 pockets prepared by MD simulation with the initial coordinate of PDBID: 4I5I. Top-ranked compounds in the ensemble docking were collected, and their docking poses were produced. The docking poses were visually presented to volunteer voters. The voters conducted visual inspection, and selected “good,” “bad,” or “no idea” for each of the docking poses. Finally, the compounds were reranked by the vote result.

#### Group 14 (G14)

Three methods were employed: MTS (structure-based),^36^ machine-learning MTS (ML-MTS) (hybrid of structure-based and ligand-based),^50^ and docking score index (DSI) (ligand-based)^51^ methods using myPresto. A target protein structure (PDB ID: 4ZZI) was used in MTS and ML-MTS methods. Eight known inhibitors including Splitomicin and Cambinol were used in the ML-MTS and DSI methods. Calculations were performed for the compound library included in a ready-to-dock compound database, LigandBOX^52^.

#### Group 15 (G15)

In the process of seeking inhibitors of the target protein, an LBSV method, VS-APPLE^53,54^, was used. While conventional ligand-based VS methods adopt single ligand as a template, VS-APPLE adopts the multiple-ligand template, which is composed of multiple ligands bound to the same pocket, as a template. Three ligands of the target protein were chosen and then each of them was used as a single template for independent VS. This procedure was performed, because only a few known protein-ligand complexes were available for the target in PDB and these ligands bind to different part of the binding pocket.

#### Group 16 (G16)

ECFP12^48^ fingerprints of 514 Sirtuin 1 binders downloaded from BindingDB were generated and used to build lasso, elastic net, ridge and random forest machine learning models relating *K*_i_. A protein-structure-based pharmacophore was generated as an alternative approach. The machine learning and pharmacophore models were applied to ECFP12 fingerprints of the compound library to predict *K*_i_ values. Prioritized 200-300 compounds from each of the methods above, together with hits from the similarity search using known active Sirtuin 1 binders were prepared. This protocol resulted in approximately 2,000 compounds. Finally, rescoring and clustering based on the docking of the compounds to the target protein (PDBID: 4ZZI) using GOLD were used to prioritize 400 representative compounds.

## Screening of Compounds

### Screening of potential inhibitors

**Figure 1:**
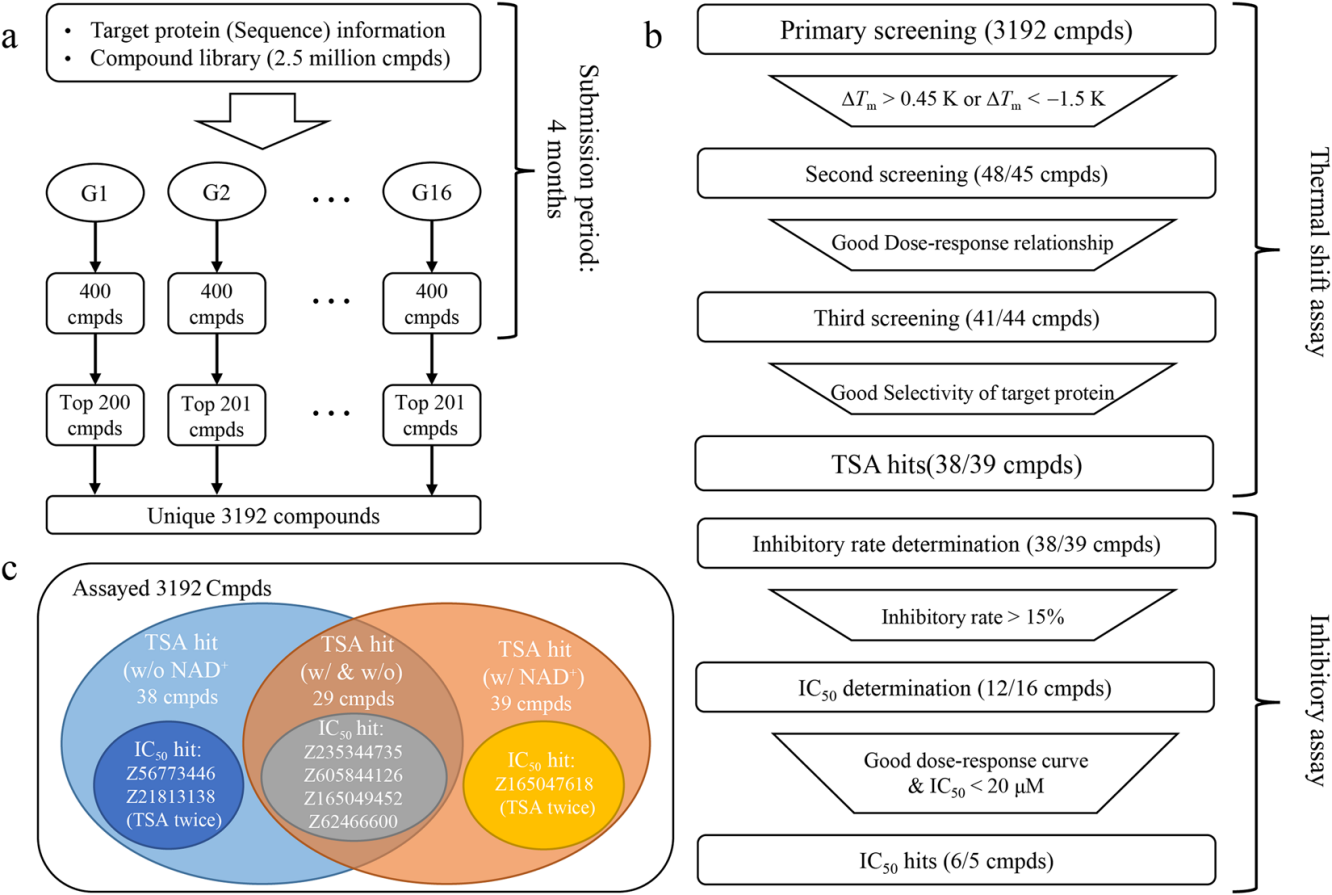
(**a**) A flowchart of the contest. Each group (G1-G16) proposed 400 compounds (cmpds) with a prioritized rank from compound library using their own methods. The proposed compounds that were not stocked-out were selected until the number of compounds reached 200 for each group. If there was duplication in the proposed compounds among different groups, these groups attained additional compounds to be assayed. For this reason, there are differences among the number of selected compounds of each group. Finally, the selected compounds were assayed. (**b**) The screening flow of the compounds in the experimental assay. The filtering criteria are shown in a trapezium. The number of compounds applied to each screening is shown in parenthesis. This flow was conducted twice with NAD^+^ (the first number in parenthesis) and without NAD^+^ (the second number in parenthesis). (**c**) IC_50_ hits found based on TSA screening without (w/o) and with (w/) NAD^+^ (see Screening of potential inhibitors in the main text).

Selection of compounds for assaying from the proposed lists and the flow of the assays are described in Fig. 1a,b. The experimental procedures are described in the supporting information in detail. Here, we briefly describe the screening flow and results.

A total of 3,192 compounds extracted from the proposed lists were first screened by thermal shift assay (TSA), which determined a melting temperature shift (Δ*T*_m_) of Sirtuin 1 upon the addition of a test compound at a concentration of 10 µM (*n* = 1) with primary hit criteria of Δ*T*_m_ > 0.45 K or Δ*T*_m_ < −1.5 K. Primary hits (48 compounds) were retested in the same assay at three concentrations (5, 10, 20 µM, *n* = 4) for hit confirmation and for dose-dependency check. Secondary hits (41 compounds) were checked their selectivity for Sirtuin 1 based on counterscreening on unrelated protein targets (bovine carbonic anhydrase (CA) and recombinant SH2 domain of hABL1 kinase (ABL1)) at three different concentrations (5, 10, 20 µM, *n* = 4), which yielded 38 hit compounds, hereafter referred to as TSA hits. The TSA screening above was conducted in the absence of NAD^+^. As noted, the structure of Sirtuin 1 is different in the presence of NAD^+^ and substrate. The chemical profile of TSA hits found from the different conditions could be different. Hence, we applied the same TSA in the presence of NAD^+^ to the 3,192 compounds. As a result, 39 TSA hits were found. Regardless of the presence of NAD^+^, 29 compounds were shared in both TSA hits.

The 48 ( = 38 + 39 −29) unique TSA hits were evaluated by an inhibitory assay, which determined inhibition rates of enzymatic activity of Sirtuin 1 of the test compounds at a concentration of 10 µM (*n* = 4) with a hit criterion of an inhibitory rate >15%. Note that the inhibitory assay was conducted with NAD^+^. As a result, 20 hit compounds, 12 from TSA without NAD^+^ and 16 from TSA with NAD^+^, were found. An IC_50_ determination of the 20 hits was conducted for dilutions of 100 μM to 0.05 μM (*n* = 4). We identified seven compounds satisfying the hit criteria of IC_50_ < 20 μM with clear dose-response relationship as shown in Figure S6 of the supporting information. The chemical structures of the seven hits are given in Table 2.

**Table 2:** IC_50_ values of hit compounds^*a*^ and their similar known inhibitors.

Compound ID	Chemical Structure^*b*^	IC_50_ μM	95% CI μM		Group	Similar compound ID & Chemical structure	Similarity^*c*^ & Inhibition
			lower	upper			
Z56773446		4.1	1.9	8.9	6 (LB)	CHEMBL260149 (Compound 4b)^57^	0.95 (Inh. rate@25 μM: 50%)^57^
Z62466600^*^		7.1	3.0	17	6,12,13 (LB)	CHEMBL1714005 (Compound 18),^58^ CHEMBL1483080 (Compound 3)^59^	0.78 (IC_50_: 6 μM)^58^ (IC_50_: 5.9 μM)^59^
Z235344735		7.6	6.4	9.1	15 (Hybrid)	CHEMBL449117	0.64 (Inhibition rate@200 μM: 28%)^69^
Z21813138		7.7	6.4	9.3	3 (LB)	CHEMBL2436120	0.65 (Inh. rate@40 μM: 70%)^70^
Z165049452		12	9.3	14	13 (Hybrid)	CHEMBL2332048	0.68 (IC_50_> 50 μM ^*d*^)^35^
Z165047618^*^		13	7.6	23	9 (SB)	CHEMBL1762050	0.60 (Inh. rate@50 μM = 70%)^71^
Z605844126		15	10	22	1 LB->SB	CHEMBL3222149	0.65 (Inh. rate@100 μM = 10%)^72^

*a*) The melting temperature shift (Δ*T*_m_) of Sirtuin 1 upon addition of a test compound and the inhibition rates are shown in the Supporting Information along with the canonical SMILES. The final Sirtuin 1 concentrations used for the IC_50_ determination were 30 nM for compounds marked with * and 20 nM for the others. b) The stereochemistry of compounds was not determined. c) Similarity scores were calculated using the Tanimoto coefficients of the MACCS descriptor. d) This compound was included as a known inhibitor despite its weak potency, because the series of its analogues are inhibitors.^35^ IC_50_ = inhibitory concentrations; CI = confidence interval.

We first noticed that Z56773446 and Z21813138 were found only through primary screening without NAD^+^; on the other hand, Z165047618 and Z62466600 were found only through primary screening with NAD^+^. To confirm whether the results of primary screenings of Z56773446 and Z21813138 with NAD^+^ and Z165047618 and Z62466600 without NAD^+^ were false negatives or not, we conducted additional experiments of TSA on Z165047618 and Z21813138 with NAD^+^ (*n* = 4) and Z56773446 and Z62466600 without NAD^+^ (*n* = 4). We confirmed that the Δ*T*_m_ values of Z165047618, Z21813138, and Z56773446 were not as large as the hit criterion, *i.e*., −0.03, 0.32, and −0.05, respectively. However, the Δ*T*_m_ of Z62466600 was 0.87, and its hit confirmation and dose-dependence of the three concentrations were also confirmed. Hence, we decided that the first primary screening of Z62466600 was a false negative and that it should be classified as an IC_50_ hit (w/ & w/o NAD^+^). It is not clear why results that were shown in Fig. 1c were obtained, based on the limited information of the present study.

The seven hit compounds were compared to the pan-assay interference compounds (PAINS) filters,^55^ which collects substructures of frequent hitters that appeared in many biochemical high-throughput screenings. As a result, Z21813138, Z235344735, Z605844126, Z165049452, and Z165047618 were confirmed not to have the potential problematic substructures. This result indicates that these compounds are promising candidates for further investigation. On the other hand, Z56773446 and Z62466600 were found to have potentially problematic substructures, *i.e*., Z56773446 has a divinylketone (PAINS filter entry: ene_one_ene_A), and Z62466600 contains alkylidene barbiturate (PAINS filter entry: ene_six_het_A). Despite of these potentially problematic substructures, we regarded these compounds as hit compounds, because there are known inhibitors similar to them, as shown in Table 2, and these compounds passed the counterscreening on unrelated protein targets. Note that the Δ*T*_m_ values of Z56773446 and Z62466600 at 5, 10, and 20 μM (determined without NAD^+^) were as follows: For Z56773446, Sirtuin 1: 0.4, 0.6, 1.0; CA: 0.2, 0.5, 0.5; and ABL1: 0.0, 0.1, 0.2; and for Z62466600, Sirtuin 1: 0.2, 0.3, 0.5; CA: 0.0, 0.0, 0.0; and ABL1: −0.2, 0.1, 0, respectively.

Information of the assayed compounds is given in the supporting information, including the results of Δ*T*_m_, dose-dependence check, selectivity, inhibition rates, and IC_50_ values.

## Discussion

### Hit rate of assayed compounds

Among 3,192 assayed compounds, 7 and 4 compounds showed IC_50_ values less than 20 μM and 10 μM, respectively, as shown in Table 2. To evaluate the success odds of the contest, we compared this value (4/3192) with a hit rate obtained from a previous HTS study on inhibitors against recombinant Sirtuin 1. Therein, 58 inhibitors with an IC_50_ less than 10 μM were identified from among 147,000 different compounds^56^. Thus, the success odds of the contest (0.13% = 4/3192) under the same hit definition was higher than that reported previously (0.04%). To determine whether this improvement is statistically significant, we performed an exact binomial test with a significance level of 0.05. The *p*-value of the test was 0.04 and less than the defined significance level. Although the previously applied experimental conditions and compound library were not identical to the contest, the results indicated that a contest-based approach would be successful in comparison with a simple HTS even in the hit rate. The total hit rate in this study, 7/3192, was lower than that of the previous contest, 11/1991, which targeted the tyrosine-protein kinase Yes when IC_50_ < 20 μM. This reduction in the hit rate may have resulted from a greater difficulty in identifying an inhibitor of the target molecule,* i.e*., the hit rate for HTS of Sirtuin 1 (0.04%)^56^ was lower than that for kinase (0.06%)^1^.

The most successful methods in terms of the hit rate have been proposed by G6 and G13, both of which identified two hits. The performance of these methods, relative to that of other methods, was evaluated using the binomial test. Provided that the average hit rate was 7/3192, the *p*-values of G6 and G13 were both 0.07; hence, they may not be considered promising methods relative to the other methods. Assuming that the success odds of the HTS study, 0.04%, can be considered the baseline for random screening, a hit rate of 2/181 of G6 under the hit criterion of IC_50_ < 10 μM yielded a *p*-value of 0.0025. Even with Bonferroni correction, this *p*-value indicated that the method of G6 is statistically promising (the *p*-value < 0.05/16) than the HTS.

In this study, we do not review each method in detail, but rather, we present an overview of the contest-based approach for the identification of potential inhibitors for the target molecule.

### Diversity of proposed compounds

**Figure 2:**
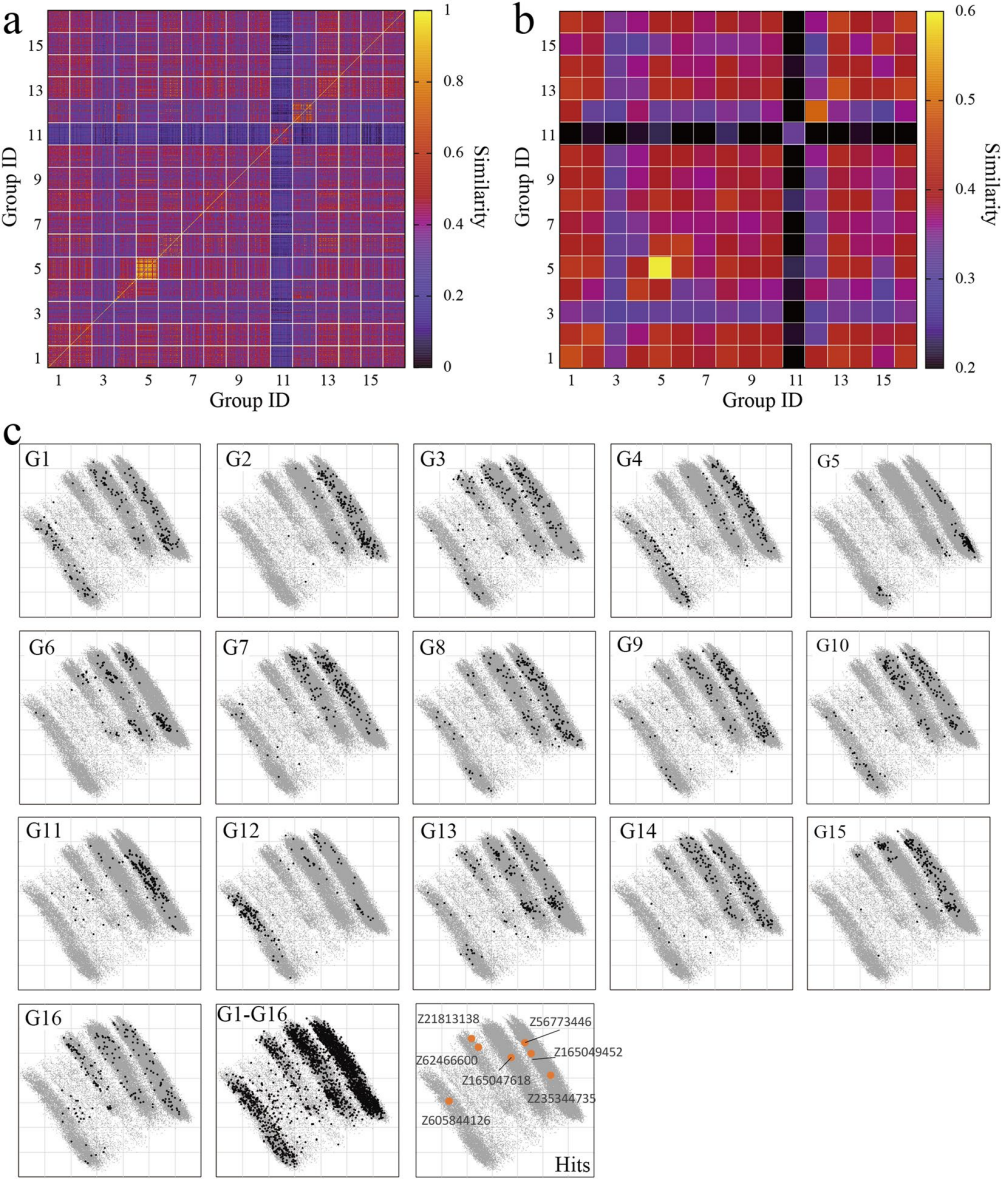
(**a**) Similarity of the compounds proposed from each group. The similarity scores are defined with the Tanimoto coefficient of the MACCS descriptor^60^. The number of identical compounds proposed from different two groups is shown in Figure S7, which indicates that identical compounds were rarely proposed from different groups, except for the combinations of G6 and G12 (19 compounds) and G6 and G13 (5 compounds), which used ligand information. (**b**) Averaged similarity scores in each cell of (**a**), in which identical compounds on the diagonal are not included for averaging. (**c**) Assayed compounds from each group are projected to the first and second principal components (PC1: x-axis, PC2: y-axis). Hit compounds are projected to PC1 and PC2 as well. Principal component analysis was applied to the compound library using the MACCS descriptor. The cumulative variance of the PC1 and PC2 are 26% and 50%, respectively. A randomly chosen 3% of the compounds in the library are projected (gray points).

We have repeatedly emphasized that the contest-based approach realized diversification in the screening results in terms of the chemical diversity of the assayed compounds.^9,10^ This observation was applicable to the present results, as shown in Fig. 2. The average similarity of the different compounds in each group, 0.44 ± 0.09 (average and standard deviation of 16 methods, diagonal elements of Fig. 2b, of which values are summarized in Table S2), was higher than the average similarity of the different compounds in all groups, 0.36 ± 0.08 (average and standard deviation of 120 + 16 combinations, off-diagonal and diagonal elements of Fig. 2b), *i.e.*, the *p*-value of Welch’s t-test with the null hypothesis that the two averages are equal was 0.005. In addition, as seen in Tables S2 and S3, we did not observe that a specific filter class mainly contribute to diversifying screened compounds.

There were two groups that showed higher diversity than the average of all groups, *i.e.*, G3 (LB approach) and G11 (SB approach), of which average diversities of assayed compounds were 0.30 and 0.33, respectively. Even though the two methods’ average diversities were higher than the average of all groups, each method typically yielded compounds from different places in the chemical space of the compound library, as visualized in Fig. 2c, indicating that the contest-based approach can realize diversified screening.

The final hit compounds were also chemically diverse, as seen in Table 2. The maximum similarity among the hit compounds was 0.54, which was the score between Z56773446 and Z62466600.

### Novelty of the assayed and hit compounds

Finding chemically novel compounds in the early stage of drug discovery process is crucially important. To evaluate the performance of the contest-based approach to find such compounds, we compared the hits to the known inhibitors of Sirtuin 1. The known inhibitors in this study were collected from ChEMBL version 20 with queries of Standard type including IC, EC, or inhibition and were then filtered by the criteria of an inhibition rate less than 0% or an IC_50_ greater than 100 μM. Removing duplicates, 892 unique inhibitors were identified. The inhibitor set included Sirtuin 1 ligands defined in the MUBD-HDACs database^15^ except a compound (ChEMBL ID CHEMBL99779). In this study, the compound was not defined as an inhibitor, because relevant information for the compound regarding inhibition of Sirtuin 1 was not found in ChEMBL and BindingDB.

First, we evaluated the average similarity of assayed compounds to the known inhibitors. The average similarities were 0.33 ± 0.02, 0.24 ± 0.06, and 0.34 ± 0.01 for LB, SB, or hybrid methods, respectively. These values were low, and each filter class proposed novel compounds on average.

**Figure 3:**
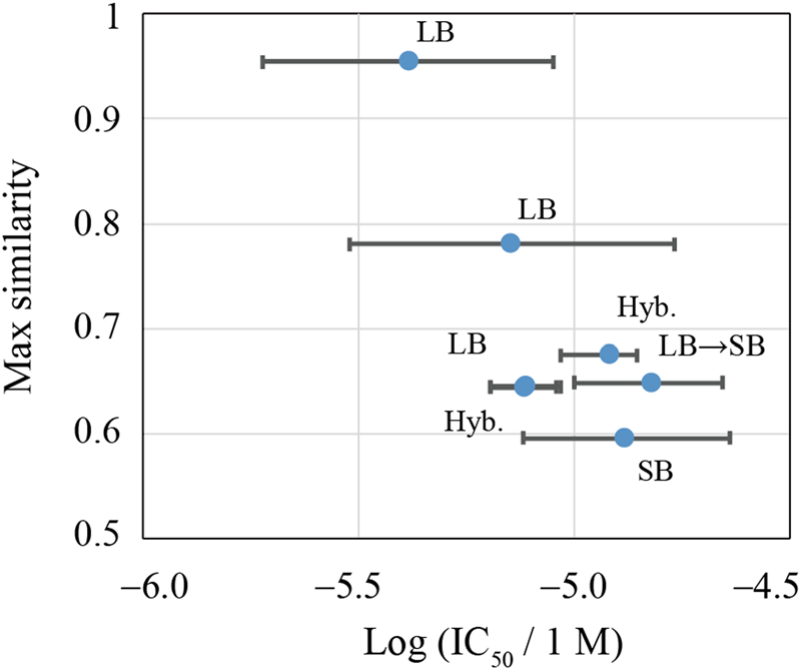
The similarity of each hit compound to known Sirtuin 1 inhibitors (see Novelty of the assayed and hit compounds Section) is plotted against the experimental inhibition activity. The error bar represents 95% confidence intervals estimated from IC_50_ assays. For each point, the category of method used is presented (see Table 1). The similarity in the figure was calculated with the Tanimoto coefficient of the MACCS descriptor^60^.

Thereafter, we investigated similarities among hit compounds with the known inhibitors. We regard that Z235344735, Z21813138, Z165049452, Z165047618, and Z605844126 are novel, because their maximum similarity to known compounds was 0.68, and comparison of the chemical structures between a hit and the corresponding inhibitor were not similar as shown in Table 2. Among them, four compounds were proposed from methods that use ligand information (LB, hybrid, LB- > SB methods) (G1, G3, G13, G15), and one compound, from the SBVS method (G9). This means that the use of ligand information does not necessarily yield similar compounds to known inhibitors. The most novel compound (Z165047618) was proposed from SBVS. However, the potency of the hit was classified as weak among the seven hits, as shown in Fig. 3. In the previous study, novel but weak hits were also found from SBVS^10^. Hence, novelty and potency tend to display a tradeoff association.

The remaining two compounds were similar to the known inhibitors, as shown in Table 2. The similar known compound of Z56773446 was reported as compound 4b^57^ (Tanimito coefficient of the MACCS descriptor was 0.95). In the literature, the authors evaluated the effect of 4b on apoptosis induction and granulocytic differentiation in the human leukemia U937 cells and found high differentiating activity. Hence, cell-based assay of Z56773446 is considered interesting. Similar known compounds of Z62466600 were reported as compound 18^58^ and compound 3^59^ in two studies (both Tanimoto coefficients were 0.78). Compound 18 was derived from optimizing Cambinol, a sirtuin inhibitor that shows antitumor activity in preclinical models. Compound 18 showed selectivity for Sirtuin 1 (Sirtuin 1 IC_50_ = 6 μM, and Sirtuin 2 IC_50_ = 20 μM). Compound 3 showed selectivity for Sirtuin 1 (Sirtuin 1 IC_50_ = 5.9 μM, Sirtuin 2 IC_50_ = 20.3 μM, Sirtuin 3 inhibition rate = 14% at 50 μM, Sirtuin 5 IC_50_ = 46.5 μM). Hence, further investigation of Z62466600 would be valuable.

## Conclusion

A compound screening contest was conducted to identify inhibitors of Sirtuin 1. Seven inhibitors were identified with highly diverse structures. Assuming that the hit rate of a reported HTS study^56^ is potentially applicable as a baseline for random screening, the contest-based approach could be considered significantly better than the HTS. The diversity could be attained by the collection of various methods in this approach. This observation was consistent with the previous contests. We speculated that prospective benchmarking of various methods based on the identical conditions (the compound library, experimental conditions, time period of proposal) would enable identifying a promising method for finding inhibitors of the target molecule. However, none of the proposed methods were better than the others on comparing them on the basis of the average hit rate determined from all methods, probably because of increased difficulty in identifying a novel inhibitor for Sirtuin 1 rather than the target of the previous contest, wherein a promising method was reported. Under these circumstances, an increase in the number of compounds assayed from a group would help identify a promising method. Furthermore, on comparing the proposed methods with low hit rates, consideration of not only hit rates but also potency of hits could be useful.

## Supplementary information

Supporting Information

Dataset 1

Dataset 2
