# Supplementary material for: A prospective compound screening contest identified broader inhibitors for Sirtuin 1
Source: Sci Rep. 2019 Dec 20;9:19585. doi: 10.1038/s41598-019-55069-y (PMC6925144; doi:10.1038/s41598-019-55069-y)
Supplement: Supplementary file 1 — Supporting Information [file 41598_2019_55069_MOESM1_ESM.pdf]

Supporting Information for

A prospective compound screening contest identified broader inhibitors for Sirtuin 1

Shuntaro Chiba[1,3,4], Masahito Ohue[2,3], Anastasiia Gryniukova[5], Petro Borysko[5], Sergey Zozulya[5], Nobuaki Yasuo[2,7], Ryunosuke Yoshino[1,3,37], Kazuyoshi Ikeda[6], Woong-Hee Shin[8], Daisuke Kihara[8,9], Mitsuo Iwadate[10], Hideaki Umeyama[10], Takaaki Ichikawa[10], Reiji Teramoto[11], Kun-Yi Hsin[12], Vipul Gupta[13], Hiroaki Kitano[12,13,14], Mika Sakamoto[15], Akiko Higuchi[16], Nobuaki Miura[15], Kei Yura[15,17,18], Masahiro Mochizuki[1,19], Chandrasekaran Ramakrishnan[20], A. Mary Thangakani[20], D. Velmurugan[21], M. Michael Gromiha[3,20], Itsuo Nakane[22], Nanako Uchida[23], Hayase Hakariya[24,39], Modong Tan[25], Hironori K. Nakamura[26], Shogo D. Suzuki[2], Tomoki Ito[27], Masahiro Kawatani[27], Kentaroh Kudoh[27], Sakurako Takashina[27], Kazuki Z. Yamamoto[28], Yoshitaka Moriwaki[29], Keita Oda[30,40], Daisuke Kobayashi[31], Tatsuya Okuno[32], Shintaro Minami[33], George Chikenji[31], Philip Prathipati[34], Chioko Nagao[34], Attayeb Mohsen[34], Mari Ito[34], Kenji Mizuguchi[34], Teruki Honma[2,3,35], Takashi Ishida[1,2,3], Takatsugu Hirokawa[36,37,38], Yutaka Akiyama[1,2,3,38], Masakazu Sekijima[1,2,3,38*]

[1]Education Academy of Computational Life Sciences (ACLS), Tokyo Institute of Technology, 4259 Nagatsutacho, Midori-ku, Yokohama 226-8501, Japan

[2]Department of Computer Science, School of Computing, Tokyo Institute of Technology, 2-12-1 Ookayama, Meguro-ku, Tokyo 152-8550, Japan

[3]Advanced Computational Drug Discovery Unit, Tokyo Institute of Technology, J3-23-4259 Nagatsutacho, Midori-ku, Yokohama 226-8501, Japan

[4]RIKEN Medical Sciences Innovation Hub Program, 1-7-22, Suehiro-cho, Tsurumi-ku, Yokohama, 230-0045, Japan

[5]Bienta/Enamine Ltd., 78 Chervonotkatska Street 78, Kyiv 02094, Ukraine

[6]Faculty of Pharmacy, Keio University, 1-5-30 Shibakoen, Minato-ku, Tokyo 105-8512, Japan

[7]Research Fellow of the Japan Society for the Promotion of Science DC1

[8]Department of Biological Science, Purdue University, West Lafayette, Indiana, 47907, USA

[9]Department of Computer Science, Purdue University, Indiana, 47907, USA

[10]Department of Biological Sciences, Chuo University, 1-13-27 Kasuga, Bunkyo-ku, Tokyo 112-8551, Japan

[11]Discovery technology research department, Research division, Chugai Pharmaceutical Co.,Ltd., 200, Kajiwara, Kamakura, Kanagawa, 247-8530, Japan

[12]Okinawa Institute of Science and Technology Graduate University, 1919-1 Tancha, Onna-son, Kunigami, Okinawa 904-0495, Japan

[13]The Systems Biology Research Institute, Falcon Building 5F, 5-6-9 Shirokanedai, Minato-ku, Tokyo 108-0071, Japan

[14]Center for Integrative Medical Sciences, RIKEN, 1-7-22 Suehiro-cho, Tsurumi-ku, Yokohama City, Kanagawa, 230-0045, Japan

[15]Graduate School of Humanities and Sciences, Ochanomizu University, 2-1-1 Otsuka, Bunkyo-ku, Tokyo 112-8610, Japan

[16]Department of Computational Biology and Medical Sciences, Graduate School of Frontier Sciences, The University of Tokyo, 7-3-1 Hongo, Bunkyo-ku, Tokyo 113-8654, Japan

[17]Center for Simulation Science and Informational Biology, Ochanomizu University, 2-1-1 Otsuka, Bunkyo-ku, Tokyo 112-8610, Japan

[18]School of Advanced Science and Engineering, Waseda University, 3-4-1 Okubo, Shinjuku-ku, Tokyo 169-8555, Japan

[19]IMSBIO Co., Ltd., Level 6 OWL TOWER, 4-21-1 Higashi-Ikebukuro, Toshima-ku, Tokyo 170-0013, Japan

[20]Department of Biotechnology, Bhupat Jyoti Mehta School of Biosciences, Indian Institute of Technology Madras, Chennai, 600036, Tamilnadu, India

[21]CAS in Crystallography and Biophysics and Bioinformatics Facility, University of Madras, Chennai, 600025, Tamilnadu, India

[22]Okazaki City Hall, 2-9 Juo-cho Okazaki, Aichi 444-8601, Japan

[23]IQVIA Services Japan K.K., 4-10-18 Takanawa Minato-ku, Tokyo 108-0074, Japan

[24]Institute for Chemical Research (ICR), Kyoto University, Uji, Kyoto 611-0011, Japan

[25]Department of Chemistry & Biotechnology, The University of Tokyo, 7-3-1 Hongo, Bunkyo-ku, Tokyo 113-8654, Japan

[26]Biomodeling Research Co., Ltd., 1-704-2 Uedanishi, Tenpaku-ku, Nagoya 468-0058, Japan

[27]Faculty of Medicine, Akita University, 1-1-1 Hondo, Akita 010-8543, Japan

[28]Isotope Science Center, The University of Tokyo, 2-11- 16, Yayoi, Bunkyo-ku, Tokyo 113-0032, Japan

[29]Department of Biotechnology, The University of Tokyo, 1-1-1 Yayoi, Bunkyo-ku, Tokyo 113-8657, Japan

[30]Google Japan Inc., 6-10-1 Roppongi, Minato-ku, Tokyo 106-6126, Japan

[31]Department of Computational Science and Engineering, Nagoya University, Furocho, Chikusa-ku, Nagoya 464-8603, Japan

[32]Tosei General Hospital, 160 Nishioiwake-cho, Seto, Aichi 489-8642, Japan
[33]Department of Complex Systems Science, Graduate School of Information Science, Nagoya University, Furocho, Chikusa, Nagoya, 464-8601, Japan
[34]National Institutes for Biomedical Innovation, Health and Nutrition, Osaka, 567-0085, Japan
[35]Center for Life Science Technologies, RIKEN, 1-7-22 Suehiro, Tsurumi-ku, Yokohama, Kanagawa, 230-0045, Japan
[36]Molecular Profiling Research Center for Drug Discovery, National Institute of Advanced Industrial Science and Technology, 2-4-7 Aomi, Koto-ku, Tokyo, 135-0064, Japan
[37]Division of Biomedical Science, Faculty of Medicine, University of Tsukuba, 1-1-1 Tennodai, Tsukuba-shi, Ibaraki, 305-8575, Japan
[38]Initiative for Parallel Bioinformatics, Level 14 Hibiya Central Building, 1-2-9 Nishi-Shimbashi Minato-Ku, Tokyo, 105-0003, Japan
[39]Training Program of Leaders for Integrated Medical System (LIMS), Kyoto University, Uji, Kyoto 611-0011, Japan
[40]Present address: Otemachi Bldg. 3F, 1-6-1, Preferred Networks, Otemachi, Chiyoda-ku, Tokyo, 100-0004, Japan

1. Experimental procedures

A. Expression and purification of human SIRT1 protein.

Recombinant truncated version of human SIRT1 enzyme was expressed in *Escherichia coli* M15 cells and purified as described below. Plasmid construct pQE-80-SIRT1 for the production of hSIRT1 was a gift from John Denu obtained through Addgene (https://www.addgene.org, Addgene plasmid #13735). Construction of the plasmid and purification of the protein was previously described.[1] The construct encodes amino acid sequence of human Sirtuin 1 (NCBI Reference Sequence: NP_036370) with amino acids residues 6 to 83 deleted and a six-histidine tag sequence introduced at the amino terminus to facilitate protein purification. As a result of cloning manipulations, the recombinant protein (689 amino acids, calculated molecular mass 76,301 Da) also contains 6 extraneous amino acid residues bracketing the His-tag and 8 additional amino acids added at the carboxy-terminus (Figure S1).

In a typical expression experiment, 20 mL of LB media containing 100 µg/mL ampicillin and 50 µg/mL kanamycin were inoculated with a single colony of *E. coli* M15 cells[2] transformed with pQE-80-SIRT1 plasmid picked up from an LB agar plate containing 100 µg/mL ampicillin and 50 µg/mL kanamycin. The culture was grown at 37 ºC with shaking at 250 rpm overnight (Innova 4330, New Brunswick Scientific). This overnight culture was used to inoculate 1 L of LB medium supplemented with ampicillin (100 mg/L) and kanamycin (50 mg/L). The culture was further grown at 37 ºC with shaking at 250 rpm to the mid log-phase (OD600=0.6), at which point the shaker temperature was lowered to 20-22 ºC and cells were incubated with 250 rpm shaking for an additional 1 h. Protein expression was induced by addition of isopropyl β-D-1-thiogalactopyranoside (IPTG) to 0.5 mM final concentration. After ~12 h of cultivation, the cells were harvested by centrifugation in 500 mL tubes using Beckman Coulter Avanti J-E Centrifuge with JA-10 rotor (7,000 g at 4 ˚C for 10 min). The cell pellet was resuspended in 50 mL of lysis buffer per 1 L culture and the cells were disrupted by ultrasonic disintegration in pulse mode (5 min pulse/5 min rest on ice, 6 repeats) using Branson sonifier 250 (duty cycle – 45%, output control setting – 3). The lysis buffer contained 25 mM Tris HCl pH 8.0, 0.5 M NaCl, 10 mM imidazole, 3 mM β-mercaptoethanol, 5% glycerol and was supplemented with the cocktail of protease inhibitors (1 mM phenylmethylsulphonyl fluoride, 10 µM leupeptin, 1 µM pepstatin, 10 µM aprotinin). After sonication, the lysate was supplemented with bovine pancreatic DNAse I (final concentration 20 µg/mL) and kept on ice for 20-30 min. Cell debris was removed from the sonicated lysate by centrifugation in 35 mL tubes at 25,000 g for 10 min at +4 ˚C using Beckman Coulter Avanti J-E Centrifuge with JA-20 rotor. Cleared lysate was sequentially passed through cellulose

acetate/cellulose nitrate membrane syringe filters (Millipore) with 1.2 μm and 0.45 μm pores to remove residual particulates before chromatography.

Chromatographic purification was carried out at +4 ˚C in a batch-wise manner. Slurry of 1.5 mL of HisPur™ Ni-NTA Resin (Thermo Scientific, Cat. 88221) equilibrated with the lysis buffer was added to the clarified cell lysate and incubated at 4 ˚C with gentle rotation for 1 h. The suspension was then loaded on a 10-mL disposable column (Clontech Takara Bio) and the resin allowed to settle by gravity prior to washing it consecutively with 10 mL of the lysis buffer and then with 20 mL of wash buffer (25 mM Tris-HCl pH 7.5, 0.3 M NaCl, 25 mM imidazole, 5% v/v glycerol and 3 mM β-mercaptoethanol). SIRT1 protein was eluted from the column with 15 mL of elution buffer (25 mM Tris-HCl pH 7.5, 0.1 M NaCl, 160 mM imidazole, 5% v/v glycerol and 3 mM β-mercaptoethanol). The fractions containing SIRT1 were identified by 10% SDS-PAGE analysis, combined and concentrated to 6 mL using centrifugal ultrafiltration with a molecular weight cutoff of 50,000 (Amicon Ultra Centrifugal Filter Device) according to the manufacture's instruction. The concentrated protein pool was subjected to gel-filtration chromatography (Bio-Rad, Econo-Pac 10 DG desalting columns, 3 mL load per column) and collected in the final storage buffer (20 mM Tris-HCl pH 7.5, 100 mM NaCl, 10% Glycerol, 5 mM dithiothreitol). Then the concentration of purified protein was determined spectrophotometrically using the theoretical extinction coefficient at 280 nm (40340 $M^{-1} cm^{-1}$). The final preparation was analyzed by 10% SDS-PAGE (Figure S2) and stored at −80 ˚C in 1 mL aliquots after flash-freezing in liquid nitrogen. In agreement with the published technical data from various commercial suppliers of recombinant SIRT1 (R&D Systems, Origene, Active Motif, Lifespan Biosciences) the protein has an aberrantly low mobility in SDS-PAGE migrating between 100 kDa and 140 kDa protein markers.

```
        10         20         30         40         50         60         70         80         90        100
MRGSHHHHHH GSMADEAAAA GGEQEAQATA AAGEGDNGPG LQGPSREPPL ADNLYDEDDD DEGEEEEEAA AAAIGYRDNL LFGDEIITNG FHSCESDEED

       110        120        130        140        150        160        170        180        190        200
RASHASSSDW TPRPRIGPYT FVQQHLMIGT DPRTILKDLL PETIPPPELD DMTLWQIVIN ILSEPPKRKK RKDINTIEDA VKLLQECKKI IVLTGAGVSV

       210        220        230        240        250        260        270        280        290        300
SCGIPDFRSR DGIYARLAVD FPDLPDPQAM FDIEYFRKDP RPFFKFAKEI YPGQFQPSLC HKFIALSDKE GKLLRNYTQN IDTLEQVAGI QRIIQCHGSF

       310        320        330        340        350        360        370        380        390        400
ATASCLICKY KVDCEAVRGD IFNQVVPRCP RCPADEPLAI MKPEIVFFGE NLPEQFHRAM KYDKDEVDLL IVIGSSLKVR PVALIPSSIP HEVPQILINR

       410        420        430        440        450        460        470        480        490        500
EPLPHLHFDV ELLGDCDVII NELCHRLGGE YAKLCCNPVK LSEITEKPPR TQKELAYLSE LPPTPLHVSE DSSSPERTSP PDSSVIVTLL DQAAKSNDDL

       510        520        530        540        550        560        570        580        590        600
DVSESKGCME EKPQEVQTSR NVESIAEQME NPDLKNVGSS TGEKNERTSV AGTVRKCWPN RVAKEQISRR LDGNQYLFLP PNRYIFHGAE VYSDSEDDVL

       610        620        630        640        650        660        670        680
SSSSCGSNSD SGTCQSPSLE EPMEDESEIE EFYNGLEDEP DVPERAGGAG FGTDGDDQEA INEAISVKQE VTDMNYPSNK SCRPAAKLN
```

Figure S1. Amino acid sequence of His-tagged, truncated form of human SIRT 1 (SIRT1.1).[1] Extraneous amino acid residues are highlighted in grey, the point of deletion of 78 amino acids is indicated by the arrow.

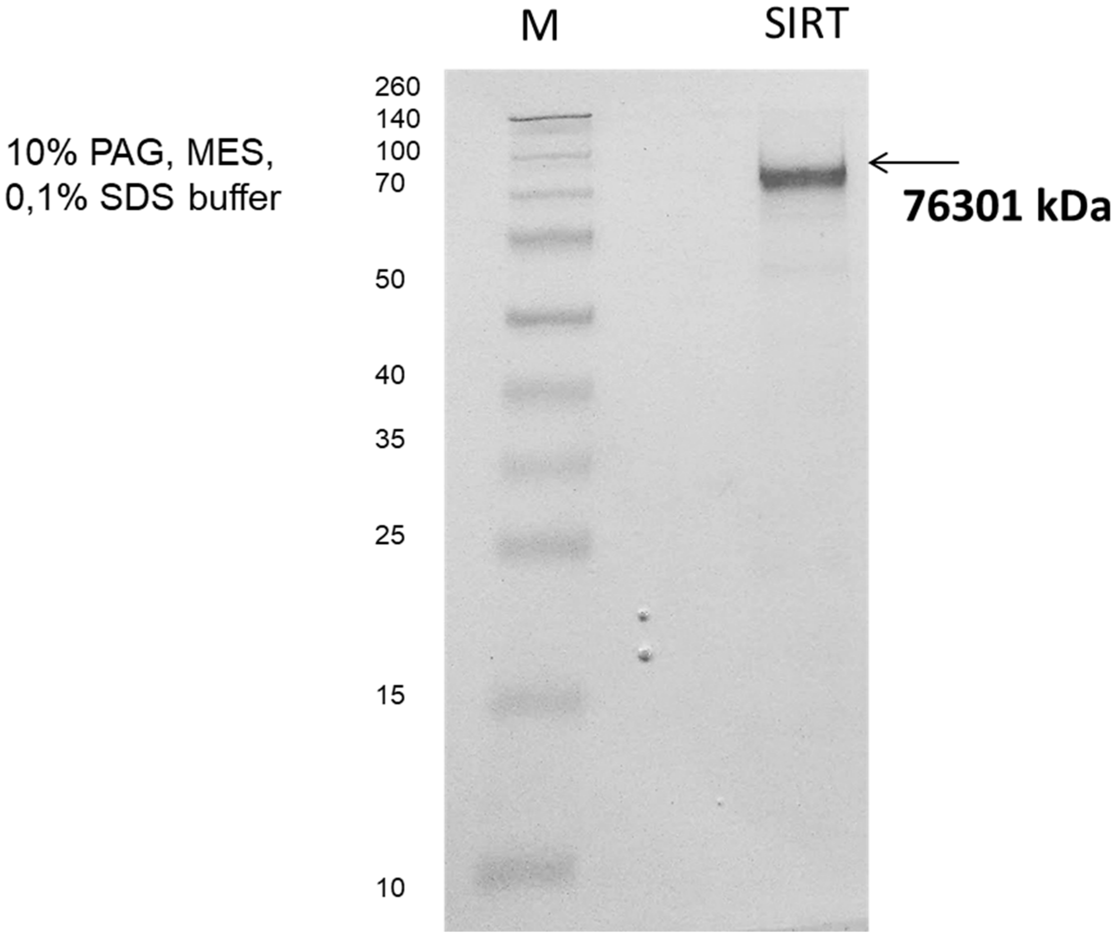

Figure S2. SDS-PAGE (reducing conditions) analysis of purified SIRT1.

B. Setup and validation of Protein Thermal Shift Assay.

All tested compounds were obtained from the screening compound collection of Enamine Ltd. (Kiev, Ukraine). Stock solutions of the tested compounds were prepared at 10 mM in 100% DMSO and were stored at –20 °C until use. All thermal shift assay (TSA) experiments with SIRT1 protein were performed using ViiA™7 real-time PCR System equipped with 384-well heat block (Applied Biosystems, USA). General TSA methodology was adopted from the literature[3-5] and experimentally modified in order to optimize conditions for measuring SIRT1 melting temperature shifts upon interaction with small molecules. To define the optimal buffer composition for the TSA procedure, a matrix of common buffers combined on a 96-well plate (Figure S3) was tested in thermal melt experiment on a 384-well plate (each buffer composition in quadruplicate). In addition, each buffer composition shown on Figure S3 was tested at two different buffering component concentrations, as listed in Table S1, resulting in two 384-well plate buffer screening experiments and the total of 192 different buffer compositions tested.

		Phosphate buffers			Acetate buffer		MES		HEPES		Tris		
					pH 4	pH 5	pH 5,5	pH 6,5	pH 7	pH7,5	pH 7,5	pH 8	pH 8,5
		1	2	3	4	5	6	7	8	9	10	11	12
**Phosphate pH 6**	A	+NaCl 50mM	+NaCl 150mM	+NaCl 300mM	+NaCl 50mM	+NaCl 50mM	+NaCl 50mM	+NaCl 50mM	+NaCl 50mM	+NaCl 50mM	+NaCl 50mM	+NaCl 50mM	+NaCl 50mM
**Phosphate pH 6,5**	B	+NaCl 50mM	+NaCl 150mM	+NaCl 300mM	+NaCl 150mM	+NaCl 150mM	+NaCl 150mM	+NaCl 150mM	+NaCl 150mM	+NaCl 150mM	+NaCl 150mM	+NaCl 150mM	+NaCl 150mM
**Phosphate pH 7**	C	+NaCl 50mM	+NaCl 150mM	+NaCl 300mM	+NaCl 300mM	+NaCl 300mM	+NaCl 300mM	+NaCl 300mM	+NaCl 300mM	+NaCl 300mM	+NaCl 300mM	+NaCl 300mM	+NaCl 300mM
**Phosphate pH 7,5**	D	+NaCl 50mM	+NaCl 150mM	+NaCl 300mM	+$CaCl_2$ 1mM	+$CaCl_2$ 1mM	+$CaCl_2$ 1mM	+$CaCl_2$ 1mM	+$CaCl_2$ 1mM	+$CaCl_2$ 1mM	+$CaCl_2$ 1mM	+$CaCl_2$ 1mM	+$CaCl_2$ 1mM
**Phosphate pH 6**	E	+$MgCl_2$ 1mM	+$MgCl_2$ 10mM	+$ZnSO_4$ 10mM	+$CaCl_2$ 10mM	+$CaCl_2$ 10mM	+$CaCl_2$ 10mM	+$CaCl_2$ 10mM	+$CaCl_2$ 10mM	+$CaCl_2$ 10mM	+$CaCl_2$ 10mM	+$CaCl_2$ 10mM	+$CaCl_2$ 10mM
**Phosphate pH 6,5**	F	+$MgCl_2$ 1mM	+$MgCl_2$ 10mM	+$ZnSO_4$ 10mM	+$MgCl_2$ 1mM	+$MgCl_2$ 1mM	+$MgCl_2$ 1mM	+$MgCl_2$ 1mM	+$MgCl_2$ 1mM	+$MgCl_2$ 1mM	+$MgCl_2$ 1mM	+$MgCl_2$ 1mM	+$MgCl_2$ 1mM
**Phosphate pH 7**	G	+$MgCl_2$ 1mM	+$MgCl_2$ 10mM	+$ZnSO_4$ 10mM	+$MgCl_2$ 10mM	+$MgCl_2$ 10mM	+$MgCl_2$ 10mM	+$MgCl_2$ 10mM	+$MgCl_2$ 10mM	+$MgCl_2$ 10mM	+$MgCl_2$ 10mM	+$MgCl_2$ 10mM	+$MgCl_2$ 10mM
**Phosphate pH 7,5**	H	+$MgCl_2$ 1mM	+$MgCl_2$ 10mM	+$ZnSO_4$ 10mM	+$ZnSO_4$ 10mM	+$ZnSO_4$ 10mM	+$ZnSO_4$ 10mM	+$ZnSO_4$ 10mM	+$ZnSO_4$ 10mM	+$ZnSO_4$ 10mM	+$ZnSO_4$ 10mM	+$ZnSO_4$ 10mM	+$ZnSO_4$ 10mM

Figure S3. Matrix plate of the buffer compositions assessed for TSA optimization.

Table S1. Buffer concentrations used for preparing buffer composition matrix screening plates.

Buffer	Plate 1 (mM)	Plate 2 (mM)
Phosphate-Na	10	100
Acetate-Na	100	200
MES-Na	10	100
HEPES-Na	10	50
TRIS-HCl	20	100

Selection of the optimal buffer composition was based on balancing several criteria including undistorted protein melting curve, maximized melting temperature stability of SIRT1 protein and maximized melting temperature shift induced by the acetylated histone H3K9 peptide (KQTARK(Ac)STGG, a natural substrate of SIRT1). Thermal shift experiments were done at 120 µM H3K9 in the presence of 500 µM NAD^+, a co-factor of SIRT1. Separate additions of H3K9 or NAD^+ at the same concentrations did not cause any thermal shift in SIRT1 melting curves. As a result, the buffer consisting of 20 mM Tris-HCl pH 8 and 50 mM NaCl was selected for the screening. Purified SIRT1 protein was pre-mixed with SYPRO Orange dye (Thermo Fischer Scientific, Cat. S6650, 5000x stock) to prepare a master mix at 400 µg/mL protein and 10x dye concentration. Tested compounds were added to the protein-dye master mix at 10 µM (1% final DMSO concentration) and incubated for 1 h at 4 °C in MicroAmp® optical 384-well reaction plates (ThermoFisher, Cat. 4309849) sealed with optical sealing film (ThermalSeal RT2, Excel Scientific, Cat. TS-RT2). The volumes of all reaction mixtures were 10 µL (4 µg SIRT1 per well). The reaction plates were then kept at ambient temperature (22-24 °C) for additional 15 minutes to ensure protein-compound interactions. Thermal scanning was performed by raising temperature to 35 °C at 1.6 °C/min without signal detection followed by 35 °C to 75 °C temperature ramp at 0.05 °C/s with constant fluorescence intensity reading at 1 sec intervals using EX470/EM623 nm filter set. Primary screening of the whole test set of 3,192 compounds was carried out in singletons. The raw data of dye fluorescence intensity change upon protein melt were exported using the ViiA7 RUO software (Applied Biosystems/ThermoFischer Scientific). Further data visualization, curve fitting, melting temperature calculations on the raw fluorescence data were performed using custom-made Microsoft Excel scripts developed by us. The peak of the first derivative for the fluorescence curve was used to define melting temperature (T_m). Averaged T_m values for the control wells (64 wells per plate), containing only the protein, dye and 1% DMSO were used as a reference point to determine melting temperature shifts (ΔT_m). Hit selection criteria was $\Delta T_m > 0.45$ °C or $\Delta T_m < -1.5$ °C. All primary hits were re-tested in the same assay at 3 concentrations (20, 10 and 5 µM, $n = 4$) for hit confirmation

and dose-dependence check (SIRT1 protein) or for a specificity counter-screen on unrelated protein targets (bovine carbonic anhydrase and recombinant SH2 domain of hABL1 kinase).

The above described procedure was also conducted without NAD^+ (see the main manuscript).

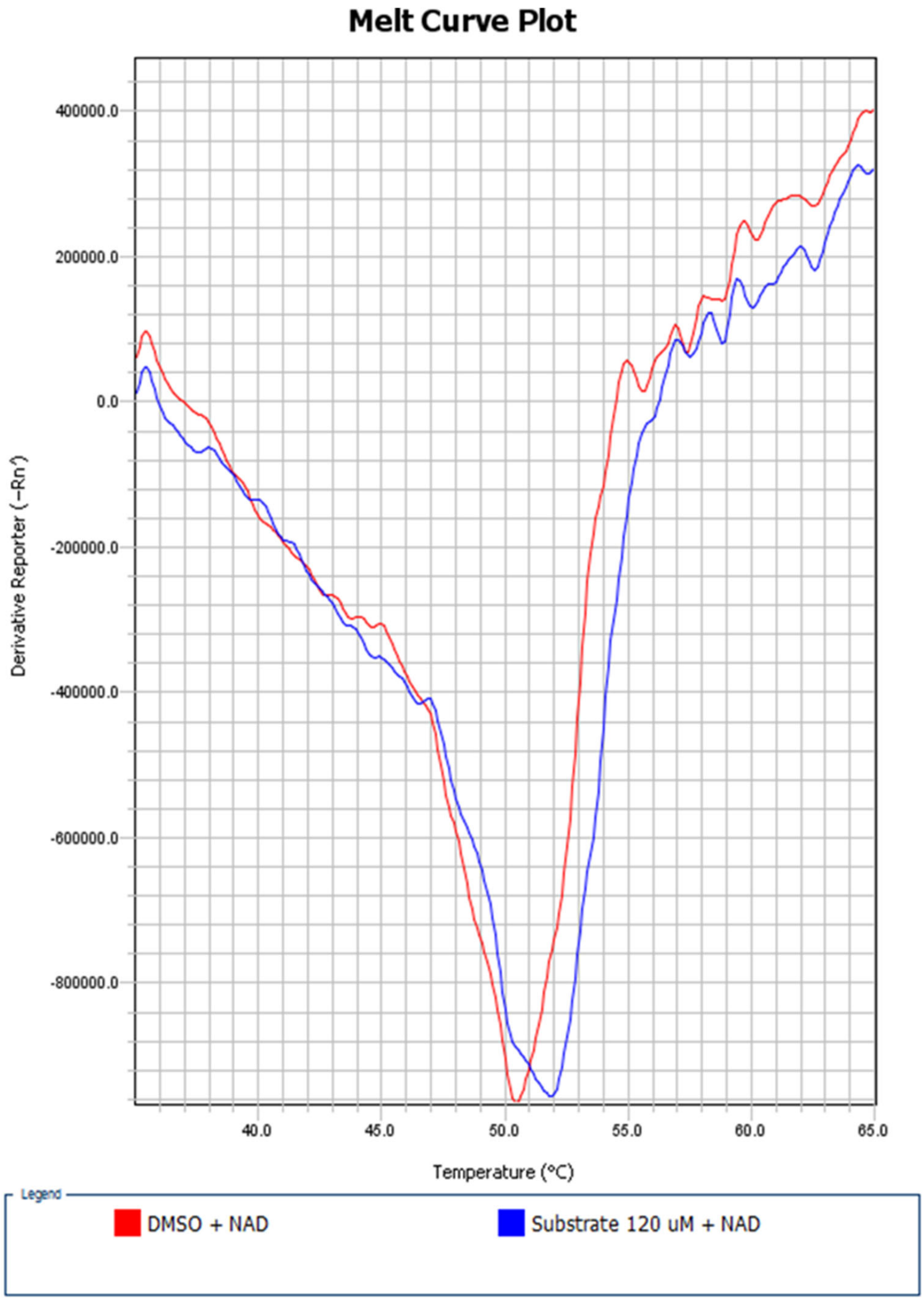

Figure S4. Typical derivative melt curve plot for SIRT1 in the absence (red line) and the presence (blue line) of histone H3K9 peptide substrate. Peptide induced thermal shift of +1.2 °C is detected.

C. Biochemical enzymatic assay for SIRT1

Luminogenic SIRT-Glo™ assay kit was purchased from Promega Corp. (Madison, WI; USA, Cat. 6450). Human SIRT1 enzyme was expressed and purified following the procedure described in the Section A of this Supplement. Enzymatic activity of SIRT1 in the presence of test compounds was assayed in 384-well white, flat bottom microplates (Thermo Scientific Matrix, Cat. 4365) using the SIRT-Glo™ assay according to the manufacturer's instructions (Promega Technical Manual for SIRT-Glo™ Assay and Screening System). Briefly, the enzymatic reactions were performed in assay buffer (25 mM Tris buffer pH 8.0, 137 mM NaCl, 2.7 mM KCl, 1 mM $MgCl_2$ and 1% Triton X100). SIRT1 was added to 3 ng/μL concentration to the wells containing tested compounds diluted in the assay buffer as well as the control wells with 1 mM nicotinamide as reference compound (1% final DMSO concentration) and the reactions (9 μL per well) were pre-incubated for 30 min at ambient temperature (22–24 °C). The enzymatic reactions were started by adding 9 μL of SIRT-Glo™ reagent (proprietary composition undisclosed by the vendor) to each assay well. After a brief vigorous mixing using VibraTranslator (Union Scientific, USA) to ensure homogeneity, the plate was incubated at the ambient temperature for 40 min. The final reaction volumes per well of a 384-well plate were 18 μL (1.5 ng/μL SIRT1: 20 nM). Luminescence signals were measured using Omega PolarStar microplate reader (BMG Labtech, Ortenberg, Germany). Luminescence values from the control plate wells with no added enzyme were used as a baseline. To validate the assay procedure, the half inhibitory concentration (IC_{50}) of a known SIRT1 inhibitor, nicotinamide, was determined (Figure S5). The resulting value was reproducible (128–252 μM) and correlated well with the value provided by Promega Corp. (89 μM) and various literature data. This validates the assay protocol and ensures that IC_{50} values may be compared between different experiments. Some compounds' IC_{50} were determined under a slightly different condition, i.e., the final SIRT1 concentration of 2.5 ng/μL (30 nM). This is because that we conducted protein expression twice and hence conducted optimization of experimental condition twice. The difference in the final concentrations would not relevant for this study because IC_{50} values determined and the final concentrations are very different.

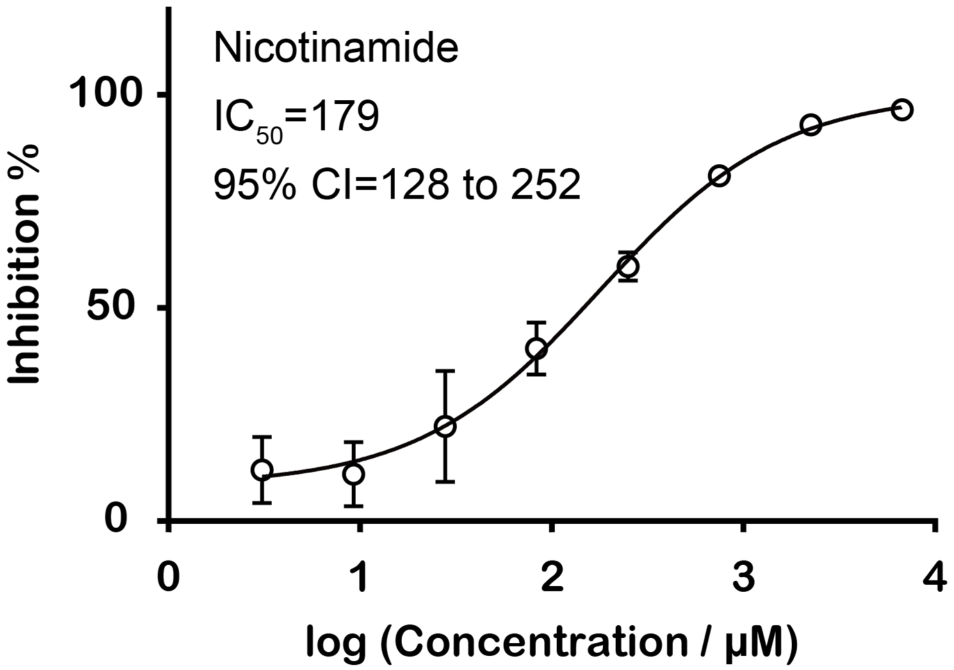

Figure S5. Dose-response curve for SIRT1 inhibition by nicotinamide under SIRT-Glo™ assay conditions described in Section C of this supporting information. The determined IC_{50} value and its 95% confidence interval are shown. The eight-point IC_{50} curve was built using 3x serial dilution starting from nicotinamide concentration of 6,750 μM. Primary compound dilutions were performed in DMSO and each concentration was tested in quadruplicate. Assay data was analyzed in GraphPad Prism,[6] using Prism's built-in sigmoidal dose-response curve-fit algorithm and default settings of the fit. Each error bar represents a standard deviation (SD) of inhibition rates for each concentration. SDs smaller than the size of circle symbol are not displayed as error bars.

D. IC_{50} determination of compounds

As described in the section Screening of compounds of the main manuscript, we conducted IC_{50} determination of 20 compounds with inhibitory rates greater than 15%. The results are shown in Figure S6.

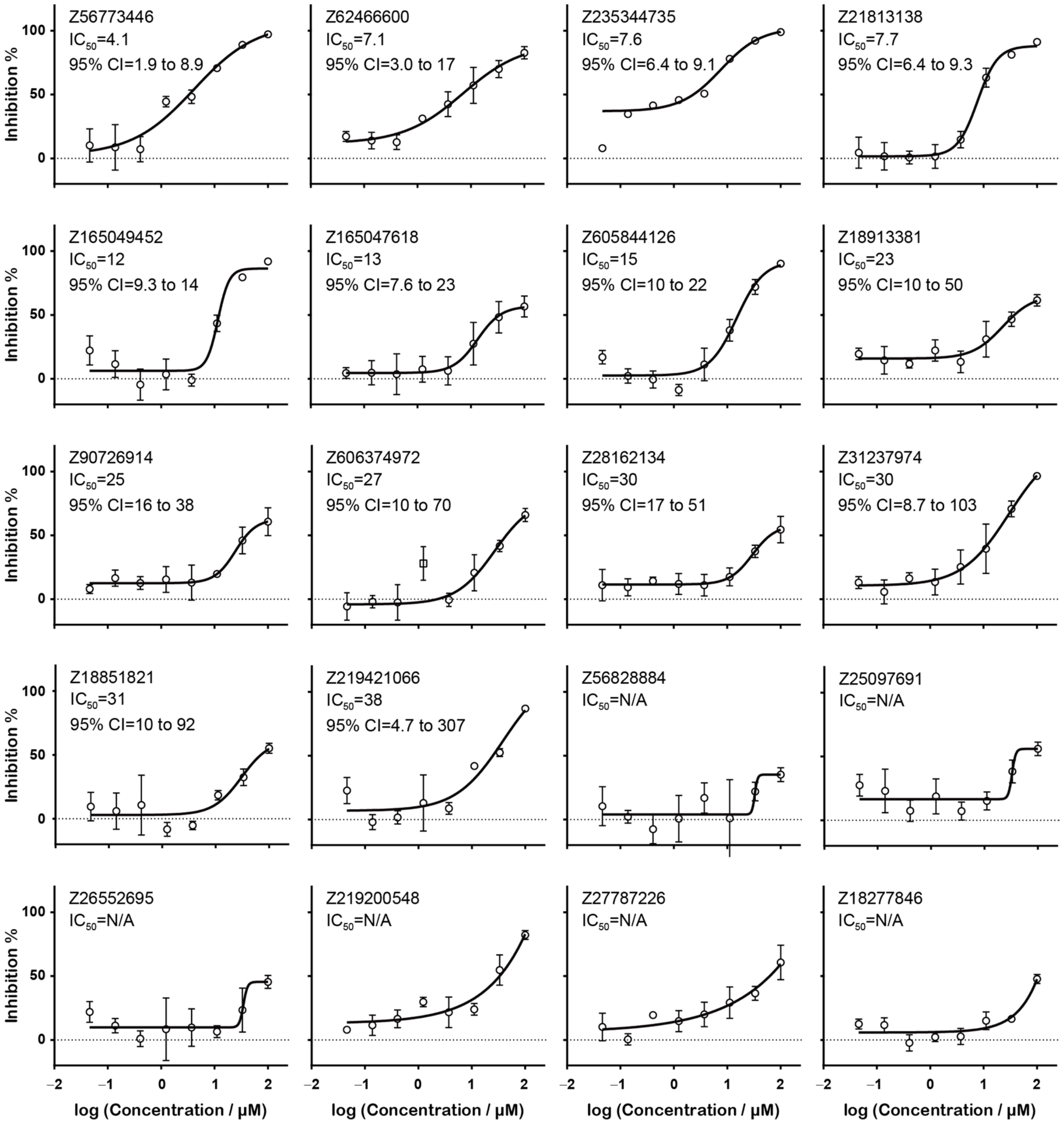

Figure S6. Dose-response curve (DRC) for SIRT1 inhibition by 20 compounds under SIRT-Glo™ assay conditions described in Section C of this supporting information. The determined IC_{50} value and its 95% confidence interval are shown. The eight-point IC_{50} curves were built using 3x serial dilution starting from compound concentration of 100 μM. Primary compound dilutions were performed in DMSO and each concentration was tested in quadruplicate. Assay data was analyzed in GraphPad Prism,[6] using Prism's built-in sigmoidal dose-response curve-fit algorithm and default settings of the fit. IC_{50} of some compounds were not available (N/A) because of an ambiguous DRC. Each bar represents a standard deviation (SD) of inhibition rates for each concentration. SDs smaller than the size of circle symbol are not displayed as error bars. Note that we removed a single data point, indicated by the square symbol, for Z606374972 from the curve-fit analysis since it is a clear

outlier. The final Sirtuin1 concentrations were 30 nM for Z90726914, Z62466600, Z165047618, Z31237974, Z219200548, Z18913381, Z27787226, Z28162134 and 20 nM for the others.

E. Average similarity of compounds proposed from each group

Table S2. Average similarity of compounds assayed from each group sorted by similarity values.

Group	Average similarity	Filter class
G3	0.30	LB
G11	0.33	SB
G7	0.37	Hybrid(LB&SB)
G9	0.39	SB
G14	0.40	Hybrid (LB&SB)
G10	0.41	Hybrid(LB&SB)
G15	0.42	Hybrid(LB&SB)
G8	0.43	Hybrid (LB→SB)
G4	0.44	Hybrid(LB&SB)
G6	0.44	LB
G16	0.45	Hybrid (LB→SB)
G2	0.45	LB→SB
G1	0.47	LB→SB
G13	0.47	Hybrid (LB,SB&visualinspection)
G12	0.49	LB
G5	0.71	Hybrid(LB&SB)

The similarity scores are defined with the Tanimoto coefficient of the MACCS descriptor.[7]

Table S3. Average similarity of compounds from LB, SB, and hybrid filter classes.

Filter class	Average similarity
LB	0.40
SB	0.36
Hybrid	0.36

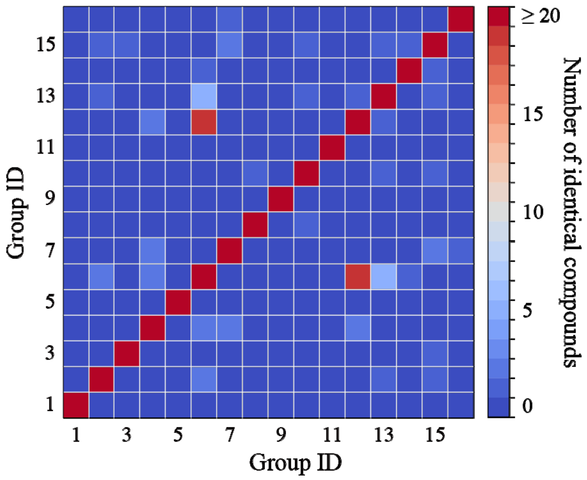

Figure S7. Identical compounds proposed from different groups.

References

1 Hallows, W. C., Lee, S. & Denu, J. M. Sirtuins deacetylate and activate mammalian acetyl-CoA synthetases. *Proc. Natl. Acad. Sci. U. S. A.* **103**, 10230-10235, doi:10.1073/pnas.0604392103 (2006).

2 Villarejo, M. R. & Zabin, I. Beta-galactosidase from termination and deletion mutant strains. *J. Bacteriol.* **120**, 466-474 (1974).

3 Lo, M. C. *et al.* Evaluation of fluorescence-based thermal shift assays for hit identification in drug discovery. *Anal. Biochem.* **332**, 153-159, doi:10.1016/j.ab.2004.04.031 (2004).

4 Matulis, D., Kranz, J. K., Salemme, F. R. & Todd, M. J. Thermodynamic stability of carbonic anhydrase: Measurements of binding affinity and stoichiometry using ThermoFluor. *Biochemistry* **44**, 5258-5266, doi:10.1021/bi048135v (2005).

5 Niesen, F. H., Berglund, H. & Vedadi, M. The use of differential scanning fluorimetry to detect ligand interactions that promote protein stability. *Nat. Protoc.* **2**, 2212-2221, doi:10.1038/nprot.2007.321 (2007).

6 GraphPad Prism v. 7.02 for Windows (GraphPad Software, La Jolla California USA).

7 Durant, J. L., Leland, B. A., Henry, D. R. & Nourse, J. G.Reoptimization of MDL keys for use in drug discovery. J. Chem. Inf. Comput. Sci. 42, 1273-1280, doi:10.1021/ci010132r (2002).
